# A γ-Secretase Inhibitor Attenuates Cell Cycle Progression and Invasion in Human Oral Squamous Cell Carcinoma: An In Vitro Study

**DOI:** 10.3390/ijms23168869

**Published:** 2022-08-09

**Authors:** Sarai Pongjantarasatian, Nunthawan Nowwarote, Varumporn Rotchanakitamnuai, Watcharee Srirodjanakul, Ritmongkol Saehun, Kajohnkiart Janebodin, Jeeranan Manokawinchoke, Benjamin P. J. Fournier, Thanaphum Osathanon

**Affiliations:** 1 Dental Stem Cell Biology Research Unit, Faculty of Dentistry, Chulalongkorn University, Bangkok 10330, Thailand; 2 Department of Oral Biology, Faculty of Dentistry, Universite Paris Cite, 75006 Paris, France; 3 Centre de Recherche des Cordeliers, INSERM UMRS 1138, Molecular Oral Pathophysiology, Universite Paris Cite, Sorbonne Universite, 75006 Paris, France; 4 Department of Anatomy, Faculty of Dentistry, Mahidol University, Bangkok 10400, Thailand; 5 Department of Anatomy, Faculty of Dentistry, Chulalongkorn University, Bangkok 10330, Thailand

**Keywords:** oral squamous cell carcinoma, Notch, g-secretase, cell cycle, Notch pathway

## Abstract

Notch signaling is associated with many human malignancies, including oral squamous cell carcinoma (OSCC). However, the exact function of Notch signaling in OSCC remains unclear. Here, we investigated the effect of Notch signaling inhibition using a γ-secretase inhibitor (DAPT) on OSCC behaviours in vitro. Bioinformatic analysis of public-available gene expression profiles revealed the dysregulation of the Notch signaling pathway in OSCC compared with normal tissues, indicating the role of Notch signaling in OSCC regulation. RNA sequencing analysis of DAPT-treated human OSCC cells revealed the dysregulation of genes related to cell cycle-related pathways. Blocking Notch signaling significantly inhibited cell proliferation. DAPT-induced G0/G1 cell cycle arrest induced cell apoptosis. Furthermore, cell migration and invasion were also reduced in DAPT-treated cells. These findings indicate that Notch signaling activation participates in OSCC regulation by promoting cell growth, cell cycle progression, cell migration, and invasion. These mechanisms could facilitate OSCC progression. These results imply the potential use of Notch signaling inhibitors as a candidate adjuvant treatment in OSCC patients.

## 1. Introduction

Oral squamous cell carcinoma (OSCC) is a common oral cancer with a high mortality rate [1]. The tongue is the most affected site, followed by the lips, oral mucosa, and gingiva [2]. Cancer initiation mechanisms in OSCC are multifactorial, including genetics, viral infection, nutrition, environment, and patient behaviors. Hence, understanding the mechanisms of OSCC development and progression is beneficial for early diagnosis, prediction, and treatment [1]. The OSCC microenvironment crucially regulates its behavior. Cells at the margin upregulate the Jak-STAT signaling pathway, which is involved in the cancer growth and survival [3,4]. The hypoxic microenvironment in OSCC regulates several cancer behaviors, including angiogenesis and invasion [5,6].

Notch signaling requires direct cell-to-cell contact since Notch receptors and ligands are transmembrane proteins. The binding of Notch ligand to its receptor results in the activation of enzymatic cleavage of receptor and further release of Notch intracellular domain (NICD). NICD then bind the transcriptional complex leading to the activation of target gene expression. Notch signaling is associated with many types of human malignancies. Notch signaling controls cell survival, proliferation, apoptosis, invasion, and metastasis in many kinds of cancer cells, for example, lung cancer cells and salivary adenoid cystic carcinoma cells [7,8]. Notch signaling pathways are related to the development and progression of many malignancies [9,10,11].

OSCC is heterogeneous in nature and composed of cancer cells in different stages of differentiation. Hence, the role of Notch signaling in OSCC remains controversial in literature since different cell stages, cancer behaviors, location, metastatic status, and aetiology could affect the function of Notch signaling in the regulation of OSCC. There is a report demonstrating that Jagged1, a canonical Notch ligand, is upregulated in OSCC [12]. NOTCH1 expression is found to be associated with the invasion and metastasis [13]. Further, NOTCH1 mutation is observed in OSCC patients [14,15]. On the contrary, another study showed the *NOTCH1* downregulation in OSCC and this involves the reduction of TNFα-induced cancer stem cells [16]. Together, the role of Notch signaling in OSCC is yet to be clarified

A previous report demonstrates that the gene expression profile from an OSCC biopsy exhibited upregulated expression of genes related to the Notch signaling [17], implying the participation of this pathway in OSCC behaviors. Expression of the Notch ligand, JAG2, is associated with a poor survival rate over a 140-month-observation period [18]. Therefore, Notch signaling is considered a potential target as adjuvant therapy in OSCC treatment. Studies have reported using a Notch signaling inhibitor as a candidate molecule for solid tumor treatment, such as pancreatic ductal and breast cancer. Clinical trials have been launched to investigate the safety and efficacy of γ-secretase inhibitors in several cancer treatments [19,20,21]. However, the molecular mechanism of the γ-secretase inhibitors in OSCC is unresolved. The present study aimed to determine the gene expression profile of γ-secretase inhibitor (DAPT) treated OSCC cells and to investigate the role of Notch signaling inhibition on OSCC cell proliferation, cell cycle progression, cell apoptosis, cell migration, and cell invasion.

## 2. Results

### 2.1. Differential Expression of Notch Signaling-Related Genes in OSCC Tissues

**Figure 1:**
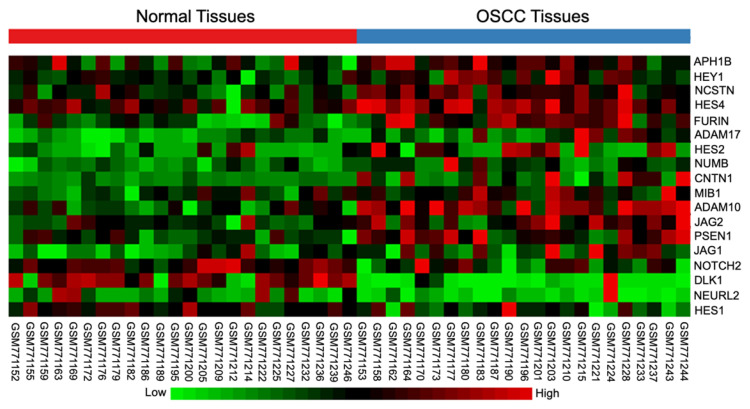
Notch signaling was dysregulated in oral squamous cell carcinoma (OSCC). A publicly available dataset of OSCC tissues and the normal oral tissues were downloaded and analysed using NetworkAnalyst [22]. Heatmap diagram showing the dysregulated genes related to Notch signaling in OSCC biopsy tissues, compared with normal tissues (normal tissues *n* = 24, OSCC tissues *n* = 23).

The differential gene expression in OSCC tissues of the tongue compared with the normal tissues was examined using bioinformatic analysis of a publicly available microarray database (GSE31056). The tissue location of those OSCC tissues of the tongue can be found in the detail of the database GSE31056. The results demonstrated that 18 Notch signaling-related genes were dysregulated in human OSCC tissues, compared to normal tissues (Figure 1). The Notch target genes *HES2*, *HES4*, and *HEY1,* were significantly upregulated, while *HES1* was downregulated. These results indicate the participation of Notch signaling in OSCC regulation.

### 2.2. Differential Gene Expression of Notch Inhibitor-Treated HSC-4 Cells

**Figure 2:**
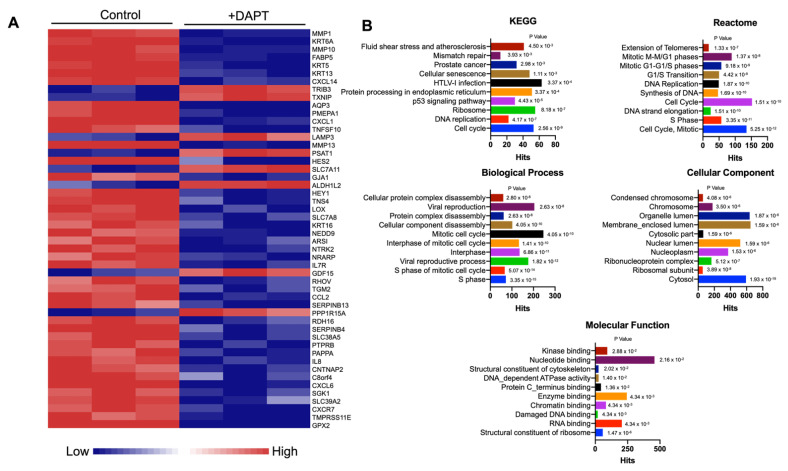
Differential gene expression analysis of a DAPT-treated human oral squamous cell carcinoma cell line (HSC-4). Cells were treated with DAPT for 24 h (*n* = 3). The differential gene expression was determined using a high throughput RNA sequencing analysis. (**A**) Heatmap of the top 50 differentially expressed genes. (**B**) The differentially expressed genes were analysed to identify related affected pathways using KEGG enrichment analysis, Reactome Pathway Database and GO terms (cellular component, molecular function, and biological process).

To examine the role of Notch signaling in OSCC, the Notch signaling inhibitor, DAPT, was employed. The HSC-4 cells were treated with 50 μΜ DAPT or 0.5% DMSO (vehicle control) for 24 h. The gene expression profile was generated using an RNA-sequencing technique, and the differential gene expression was determined using bioinformatics analysis. We found that 485 genes were differentially expressed when HSC-4 cells were treated with DAPT. Among these, 96 genes were upregulated, while 389 genes were downregulated. The top 50 differentially expressed genes are demonstrated in Figure 2A.

Bioinformatic analysis was used to identify the functional pathways of the differentially expressed transcripts. The KEGG pathway enrichment analysis revealed that the most dysregulated genes mainly participated in the cell cycle, DNA replication, ribosome, and p53 signaling pathways (Figure 2B and Table S2). Using the Reactome Pathway Database, the dysregulated genes were significantly related to the cell cycle, mitotic, S Phase, and DNA replication pathways (Figure 2B and Table S3). GO analysis showed that the downregulated genes were significantly enriched in the S phase—and cytosol-related genes in biological process and cellular component terms, respectively (Figure 2B and Tables S4 and S5). Furthermore, the structural constituents of the ribosome, RNA binding, and damaged DNA binding were significantly involved in molecular functions (Figure 2B and Table S6).

To further investigate the role of Notch inhibition in other cancers, the gene expression omnibus of breast cancer (GSE82298) and lung cancer (GSE38054) cells treated with a Notch inhibitor were downloaded and analyzed. The dysregulated genes in the Notch inhibitor-treated condition were enriched using the Reactome database. The results demonstrated that the commonly dysregulated pathways in the Notch inhibitor-treated breast cancer cells and lung cancer cells were DNA replication, cell cycle pathway, immune system, and others highlighted in yellow (Figures S2 and S3).

### 2.3. DAPT Downregulated Genes in the Cell Cycle and DNA Replication Pathways in HSC-4 Cells

**Figure 3:**
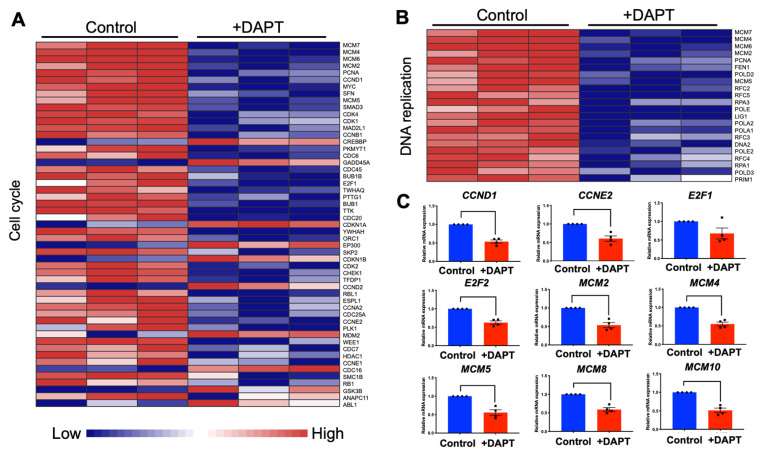
DAPT treatment reduced the expression of genes related to the cell cycle and DNA replication. (**A**) Heatmap of differential expressed genes in cell cycle pathway (*n* = 3). (**B**) Heatmap of differentially expressed genes in the DNA replication pathway (*n* = 3). (**C**) The mRNA expression levels of selected genes were determined by qRT-PCR (*n* = 4). Bars represent a significant difference between groups (*p <* 0.05).

Our analyzed RNA sequencing results indicated that the significantly downregulated genes were predominantly in the cell cycle and DNA replication pathways. Heatmaps of the dysregulated genes in the cell cycle and DNA replication pathways were prepared (Figure 3A,B). Most genes in the cell cycle pathway were downregulated, while eleven genes were upregulated, *CREBBP*, *GADD45A*, *CDKN1A, EP300, CDKN1B, CCND2, MDM2, CDC16, GSK3B, ANAPC11*, and *ABL1*. Genes in the DNA replication pathways were downregulated. The expression of selected genes was validated using qRT-PCR. The results demonstrated that *CCND1, CCNE2, E2F1, E2F2, MCM2, MCM4, MCM5, MCM8*, and *MCM10* mRNA levels were decreased after the cells were treated with DAPT for 48 h (Figure 3C). However, there was no significant difference for *E2F1*.

### 2.4. HSC-4 Proliferation Was Attenuated by DAPT Treatment

**Figure 4:**
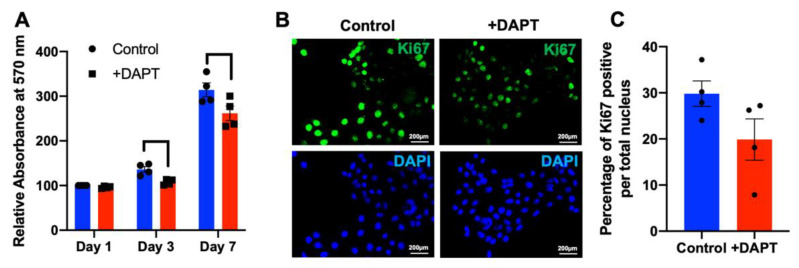
DAPT attenuated HSC-4 cell proliferation. Cells were treated with DAPT (*n* = 4). DMSO was used as the vehicle control. (**A**) Cell proliferation was determined using an MTT assay. (**B**,**C**) Ki67 protein expression was examined using immunofluorescence staining. Bars indicate a significant difference between groups (*p <* 0.05).

To explore the effect of a γ-secretase inhibitor on HSC-4 cell proliferation, the cells were grown and treated with 50 µM DAPT or 0.5% DMSO control. Cell proliferation was investigated with an MTT assay. Results indicated that growth of the DAPT-treated cells was significantly inhibited on day 3 and day 7, compared with the control (Figure 4A). A similar observation was noted in HSC7 and HN22 oral squamous cell carcinoma cell line (Figure S4). Further, DAPT treatment reduced the number of Ki67 positive cells, compared with the control (Figure 4B,C).

**Figure 5:**
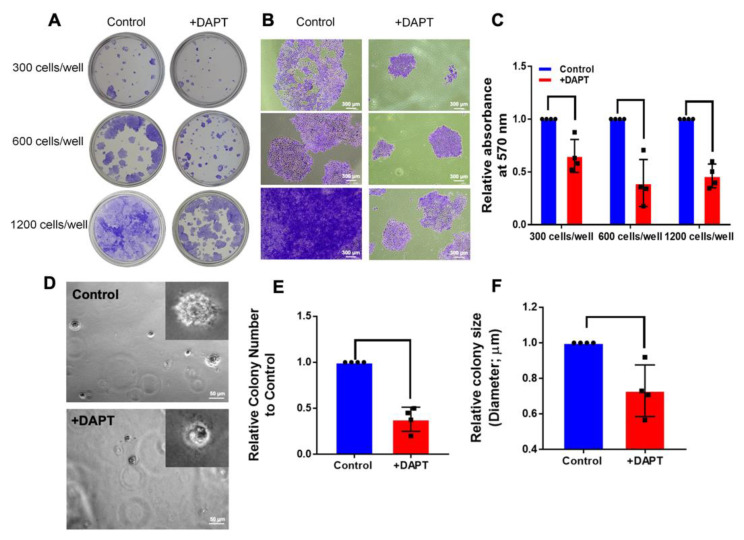
Notch signaling inhibitors inhibited colony formation in both an anchorage-dependent and anchorage-independent manner. Cells were grown in 24-well plates with various cell numbers and treated with DAPT for 14 days (*n* = 4). The colonies were stained using 0.1% crystal violet. (**A**) The overall field of colonies was taken by a digital camera. (**B**) Colony sizes were observed using a microscope. (**C**) The staining was solubilised, and the absorbance was measured at 570 nm. For anchorage-independent growth, HSC-4 cells were plated into soft agar on 24-well plates and cultured with 0.5% DMSO (Control) or 50 µM DAPT. After 14 days, the colonies were observed and photographed using a microscope. (**D**) The images show overall cell growth and sizes between cells treated with DMSO and DAPT. (**E**,**F**) Quantitative analysis identifies the size and number of colonies. Bars indicate a significant difference between groups (*p <* 0.05).

To perform an anchorage-dependent growth assay for testing the effect of DAPT on the ability of HSC-4 cells to grow and form colonies, HSC-4 cells were grown as a monolayer and treated with 50 µM DAPT for 14 d. We found that the colony sizes were noticeably reduced in the DAPT-treated group, compared with the DMSO control group (Figure 5A,B). The number of colonies was also quantified by measuring the absorbance of the eluted stain at 570 nm. As shown in Figure 5C, DAPT significantly inhibited the number of colonies.

An anchorage-independent growth assay has been reported as an accurate in vitro assay for detecting malignant transformation of cells [23]. Hence, this technique was used in evaluating the tumorigenic activity of HSC-4 cells after treatment with DAPT. HSC-4 cells were cultured in a layer of soft agar, mixed with a cell culture medium containing 0.5% DMSO (Control) or 50 µM DAPT for 14 d. The results revealed that colony size was reduced in the DAPT-treated cells (Figure 5D). Moreover, the size and number of cell colonies were significantly lower in the DAPT-treated cells, compared with the control group (Figure 5E,F).

### 2.5. DAPT Induced Cell Cycle Arrest and Cell Apoptosis

**Figure 6:**
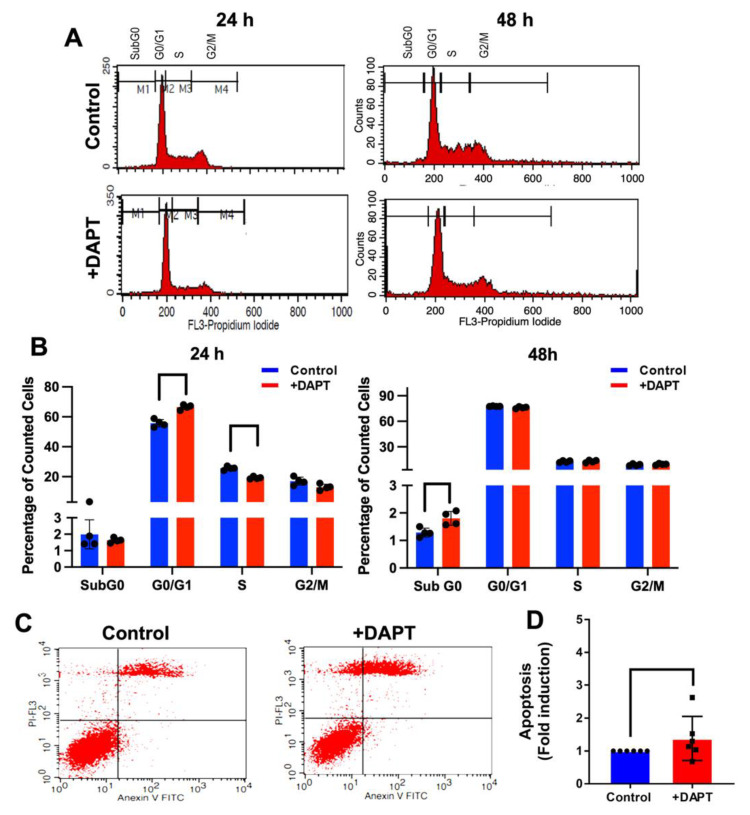
DAPT induced cell cycle arrest and cell apoptosis. Cells were treated with DAPT for 24 or 48 h. DMSO was used as the vehicle control. (**A**) The histogram shows the cell cycle progression analysed by flow cytometry. (**B**) The percentage of the cell population in the cell cycle (*n* = 4). For cell apoptosis, cells were stained with Annexin V-FITC and PI after treatment with DAPT for 48 h. The stained cells were analysed using flow cytometry. (**C**) Representative images of apoptosis were evaluated with Annexin V-FITC/PI staining in HSC4 cells. (**D**) The fold change in HSC4 cells (*n* = 6). Bars represent a significant difference between groups (*p <* 0.05).

To examine the effect of DAPT on cell cycle progression, HSC-4 cells were exposed to 50 µM DAPT or 0.5% DMSO for 24 and 48 h. The DNA content was detected using PI staining, and the cell cycle stages were analyzed by flow cytometry. At 24 h, we found that the HSC-4 cells treated with DAPT exhibited a significant increase in the cells in the G0/G1 phase population and a decreased S phase population, compared with the control (Figure 6A,B). However, the change was not a significant difference at 48 h.

The effect of DAPT on cell apoptosis was also examined. HSC-4 cells were treated with 50 µM DAPT or 0.5% DMSO for 48 h. Apoptotic cells were analyzed by flow cytometry. The results showed that DAPT treatment significantly induced apoptosis in HSC-4 cells (Figure 6C,D), corresponding with the significant increase of the Sub G0 population in the DAPT treated group, compared with the control (Figure 6B).

### 2.6. The Migration and Invasion Activities Were Abolished in DAPT-Treated HSC-4

**Figure 7:**
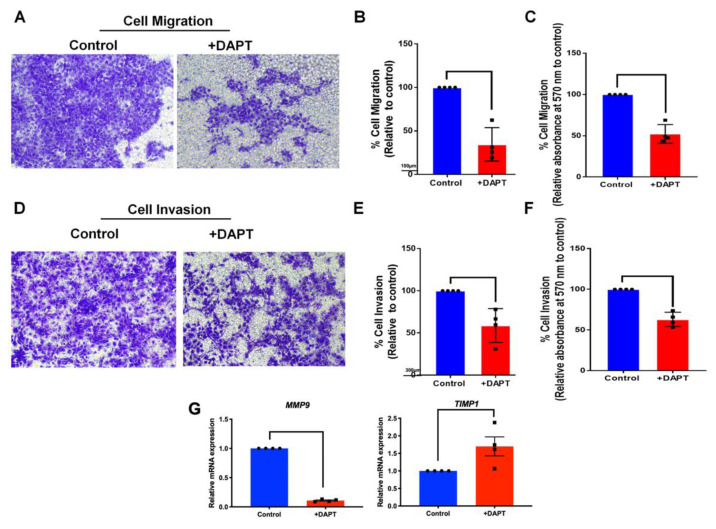
DAPT inhibited HSC-4 cell migration and invasion. The cells were treated with DAPT for 24 and 30 h for the cell migration and invasion assay, respectively (*n* = 4). (**A**,**D**) The image represents the cells that migrated or invaded the lower side of the transwell membrane. (**B**,**E**) The cell migration and invasion percentage was calculated. (**C**,**F**) The stained cells on the lower side of the transwell membrane were solubilised and the absorbance was measured at 570 nm. (**G**) The mRNA expression was examined using a real-time polymerase chain reaction. Bars indicate a significant difference between groups (*p <* 0.05).

Effects of Notch inhibition on HSC-4 cell migration and invasion were determined using transwell migration and matrigel-coated chamber assay, respectively. The result showed that inhibiting Notch signaling by DAPT treatment reduced cell migration identified by the number of migrated cells. The reduced migrated cells were noted at 48 h (data not shown). However, a significant decrease in migrated cell number was observed at 30 h (Figure 7A–C). Further, the invaded cells were significantly decreased in the DAPT treatment groups, compared with the DMSO controls at 24 h (Figure 7D–F). DAPT treatment also attenuated the *MMP9* but upregulated *TIMP-1* mRNA expression (Figure 7G).

## 3. Discussion

Notch signaling functions as an anti- and pro-proliferative regulator [24]. Previous studies showed that Notch inhibitors were essential in inhibiting cell proliferation in many cancers, including breast cancer, pancreatic cancer, and lung cancer [25]. The present study demonstrated that Notch signaling was dysregulated in OSCC biopsies. Specifically, the altered expression of the Notch target genes *HES1*, *HES4*, and *HEY1* were found. These results suggest that activating Notch signaling may contribute to OSCC pathophysiology.

The association between Notch1 expression and poor prognosis in OSCC patients has been reported [26]. Further, it has been shown that treating an OSCC cell line with DAPT significantly decreased cell proliferation in vitro [17]. DAPT at 50 μM inhibited a γ-secretase activity as it significantly reduced the expression of the Notch target gene, *HES1,* in HSC4 and HSC5 cell lines, implicating the functional activity of DAPT [17]. Correspondingly, the present study illustrated that DAPT significantly reduced cell cycle-related gene expression, for example, *CCNE2* and *CCND1* [17]. These results suggest the mechanism of Notch signaling in regulating OSCC cell proliferation.

To further investigate the role of Notch signaling in OSCC development, we also used DAPT to identify the differential gene expression in OSCC after DAPT treatment in HSC-4 cells. A previous study showed that Notch signaling was expressed in HSC-4 cells, where Notch target *HES1* and cellular proliferation were suppressed in these cells after DAPT treatment [17]. Thus, HSC-4 cells were chosen for our study, and their gene expression profile was explored by RNA sequencing.

The results of the RNA sequencing revealed that cell cycle and DNA replication were the most highly dysregulated pathways found in DAPT-treated HSC-4 cells, which correlated with the data obtained from the bioinformatic analysis of the GEO dataset of breast cancer (GSE82298) and lung cancer (GSE38054) cells. The KEGG pathway analysis revealed that cell cycle- and DNA replication-related genes were downregulated by Notch inhibitor treatment. Together, these results suggested the role of Notch signaling in regulating cancer cell proliferation.

The present study found that cell proliferation was suppressed in HSC-4 cells after DAPT treatment, which is consistent with previous studies [17]. DAPT decreased the number and size of cell colonies in both clonogenic and soft agar colony formation assays. We also found a high cell percentage in the G0/G1 phase and a reduced cell population in the S phase in the DAPT-treated cells, compared with the control. These results indicate that DAPT induces cell cycle arrest and further results in the inhibition of cell proliferation. Further, we also observed that DAPT treatment resulted in increased apoptosis in HSC-4 cells. These results are in agreement with previous studies. Inhibiting Notch signaling using a γ-secretase inhibitor in breast cancer cell lines induces G2/M arrest and triggers apoptosis [27]. The combination of DAPT and all-trans retinoic acid also inhibits cell growth and induces apoptosis in a human gastric cancer cell line [28]. In human tongue carcinoma, DAPT induced G0/G1 cell cycle arrest and apoptosis, leading to the inhibition of the cell growth [29]. The doubling time of HSC-4 is approximately 25 h, and our previous report demonstrated that the mRNA level of the proliferative marker gene (*C-FOS*) was reduced at 24 h after HSC-4 cells were treated with 50 µM DAPT [17]. We then used the time point at 24 h and 48 h for cell cycle and apoptosis assay.

Many studies demonstrated that Notch signaling affects cell proliferation by regulating the expression of cell cycle-related genes. Cyclin D1 and Cyclin E2 (encoded by *CCND1* and *CCNE2* genes) are essential regulators of cell cycle progression. These genes promote cell cycle progression in the G0/G1-S phase [30]. In embryonic stem cells, activating Notch signaling induces cyclin-D1-dependent proliferation during the neural differentiation [31]. Aberrant expression of cyclin D1 and cyclin E2 has been reported in various human cancers and correlates with the clinical outcome [32,33]. In epithelial ovarian cancer, cyclin D1 upregulation was associated with decreased patient survival [34]. The siRNA against JAG1 reduced cyclin D1 expression and the inhibition of cell cycle progression via cyclin D1-dependent G1/S checkpoint-mediated proliferation in breast cancer cells [35]. A study investigating if inhibiting the Notch pathway prevents osteosarcoma growth demonstrated that a γ-secretase inhibitor attenuated the cyclin D1, cyclin E1, E2, and SKP2 expression [36]. Another study showed that treating human cholangiocellular carcinomas xenotransplants with DAPT reduced tumor cell proliferation and induced apoptosis, while cyclin E expression was downregulated [37]. These data provided evidence that cyclin D and cyclin E are the transcriptional targets of Notch signaling, which mediates cell proliferation. Corresponding with our findings, DAPT inhibited HSC-4 cell proliferation by suppressing *CCND1* and *CCNE2* expression.

Minichromosome maintenance (MCM) family functions as a helicase required for DNA replication [38,39]. Overexpression of MCM genes is found in malignant human cancer cells [40]. In addition, pre-cancerous cells undergoing malignant transformation exhibited high expression levels of MCM genes [40]. In pancreatic cancer, the upregulation of MCM was correlated with shorter survival time [41]. In the present study, *MCM* transcript levels were suppressed in HSC-4 cells after treatment with DAPT, as shown in the RNA sequencing and qRT-PCR results, implying that Notch inhibition inhibits cell proliferation via downregulated MCM expression. Corresponding with a previous report, Notch inhibition via short-hairpin RNA (shRNA) targeting Notch2 downregulates *MCM2* and p21 expression, hence inducing cell cycle arrest in U87 human glioma cells [42]. Further investigation is indeed required to confirm this hypothesis.

The E2F family is a transcription factor regulated by the retinoblastoma protein. The E2F family is critical in controlling the cell cycle, cell proliferation, differentiation, survival, and apoptosis [43]. Moreover, the E2F family acts in both tumor suppression and oncogenesis [43]. *E2F1* is highly expressed in many cancer types [44,45]. A γ-secretase inhibitor or HES1-shRNA failed to repress the expression of *E2F1* in 3T3-L1 cells [46]. In breast cancer cells, it was shown that repressing the 17β-estradiol- and heregulin-β1-mediated up-regulation of *E2F1* was induced by HES1 [47]. These data were consistent with our results that the expression of *E2F1* was slightly decreased, but not significantly different, in the DAPT-treated cells, whereas *HES1* was significantly downregulated.

Moreover, we also found that the expression of *E2F2* was significantly suppressed in DAPT-treated cells. Similarly, a previous study revealed that cell viability and colony formation were reduced after *E2F2* expression knockdown in non-small cell lung cancer (NSCLC) cells, indicating that E2F2 acts as an activator in tumor progression of the NSCLC [48]. This evidence suggests that Notch signaling controls cell cycle progression by regulating *E2F1* and *F2F2* expression.

Notably, studies using other cancer cells demonstrated that DAPT regulates the cell cycle and influences epithelial-mesenchymal transition (EMT) and the tumor initiation [49,50]. Hence, DAPT may target OSCC via several mechanisms favouring the treatment. The role of Notch signaling in epithelial-mesenchymal-transition (EMT) has been reported. EMT was reduced in those cells treated with a γ-secretase inhibitor or knockdown of either *HEY1* or *Jagged1* expression [51,52]. Further, it is noted that hypoxia induces EMT in OSCC cell lines through Notch signaling activation, and EMT was inhibited by γ-secretase inhibitor [53]. EMT is associated with increased cancer cell migration and invasion. [54]. The present study found that DAPT significantly suppressed cell migration and invasion in HSC-4 cells, corresponding with the reduction of *MMP9* and the upregulation of *TIMP1* mRNA expression. These results confirm that Notch signaling is involved in cell migration and invasion in OSCC. Correspondingly, Notch1 overexpression promoted EMT marker expression, whereas Notch1 siRNA suppressed cell migration and invasion in prostate cancer cells [55]. Moreover, DAPT decreased Snail and vimentin expression but increased E-cadherin expression in OSCC cells [56]. This was accompanied by a reduction in the cell migration [56].

Despite evidence reported in the present study, several points need to be of concern. First, the present study employed a cell line to investigate the effect of DAPT on OSCC cells. However, the OSCC tumor is heterogeneous in nature, composed of cells in different stages and resides in the microenvironment of the complex cell interaction with other cell types, including vascular-related cells, immune cells, cancer stromal cells, and cancer stem cells. In addition, OSCC cells are also heterogeneous populations composed of cells in different stages of differentiation. Second, the aetiology of OSCC is varied considering the involvement of HPV viral infection, genetics, and environmental factors. Hence, OSCCs with different aetiology have different mechanisms regarding cancer development and progression. Therefore, the role of Notch signaling in OSCC with different aetiology is definitely distinct. Taking this information together, the interpretation and further application of Notch inhibitors in OSCC treatment should be further investigated with caution.

## 4. Materials and Methods

### 4.1. Cell Culture and Reagents

The use of a human cell line was approved by the Human Research Ethics Committee, Faculty of Dentistry, Chulalongkorn University (approval no. 017/2017). Human oral squamous cell carcinoma cells (HSC-4) were purchased from the Japanese Collection of Research Bioresources Cell Bank, National Institutes of Biomedical Innovation, Health and Nutrition, Japan. The cells were maintained in Dulbecco’s modified Eagle’s medium (DMEM) containing 10% fetal bovine serum (FBS), 100 unit/mL penicillin, 100 μg/mL streptomycin, 250 ng/mL amphotericin B, and 2 mM L-glutamine. The cells were kept at 37 °C in a humidified 5% carbon dioxide atmosphere. Culture medium and supplements were purchased from Gibco, CA, USA.

### 4.2. RNA Sequencing

The HSC-4 cells were treated with 50 µM DAPT or 0.5% DMSO (vehicle control) for 24 h. Total RNA was extracted using a RNeasy kit (Cat. No. 74104, Qiagen, MD, USA) and processed through DNase treatment. The quantity and quality of extracted RNA were examined using a Nanodrop and Aligent 2100 Bioanalyzer (Aligent, CA, USA). Bioanalyzed trace of submitted RNA was shown in Figure S1, Total RNA (200 ng) was subjected to mRNA library construction using a NEBNextÒ Ultra Directional RNA library preparation kit (New England Biolabs, Ipswich, MA, USA). Library quality was examined using an Aligent 2100 Bioanalyzer and fluorometer (DeNovix, Wilmington, DE, USA). The indexed libraries were subjected to cluster generation and sequenced on the Illumina NextSeq500 sequencer (75 bp single-end). Read quality was checked, trimmed, and filtered by FastQC and Trim Galore software [57] and subsequently mapped to the human reference genome (GRCh38) using a HISAT2 transcript aligner [58,59,60]. Differential mRNA expression analysis was determined using an edgeR package [61,62]. The raw sequencing data were submitted to NCBI’s Gene Expression Omnibus (GEO accession number GSE148919). Pathway enrichment was performed using a comprehensive network visual analytics platform for gene expression analysis, NetworkAnalyst [22]. KEGG, Reactome, and gene ontology (GO) databases were employed in the enrichment analysis [63,64,65,66,67,68,69,70,71].

### 4.3. Bioinformatic Analysis

A publicly available microarray database in Gene Expression Omnibus (GEO) was downloaded (GSE31056, GSE82298, and GSE38054) [72,73], and the expression levels were re-platformed in excel files. The gene expression dataset from normal and tumor tissues was analyzed using NetworkAnalyst [22]. The genes related to Notch signaling were listed based on a previous publication, [74]. The significant differential gene expression of Notch-related genes was determined using a defined custom signature function. A significant difference was identified when FDR < 0.05. Moreover, a heatmap of dysregulated genes related to Notch signaling was generated.

### 4.4. Real-Time Polymerase Chain Reaction (qRT-PCR)

The total RNA was extracted with a Trizol reagent (RiboExTM solution, GeneAll, Seoul, Korea). The extracted RNA quality and quantity were evaluated using Nanodrop. cDNA was obtained by converting RNA using a reverse transcriptase kit (ImProm-IITM Reverse Transcription System, Promega, Madison, WI, USA). The qRT-PCR reaction was conducted in a CFX connect Real-Time PCR (Bio-Rad, Singapore) with FastStart Essential DNA Green Master (Roche Diagnostic, Mannheim, Germany). The expression values were normalized to 18S, and the relative expression was normalized against the control. The specific primer sequences are shown in the Table S1.

### 4.5. Cell Proliferation Assay

Cells (12,500 cells/well) were plated in a 24-well plate. The cells were treated with 50 µM DAPT or 0.5% DMSO (vehicle control). On day 1, 3, and 7, MTT solution (0.5 mg/mL, 300 μL) was added to the medium and incubated at 37 °C for 30 min. Samples were washed twice with sterile phosphate-buffered saline (PBS). The formazan crystals were dissolved using DMSO and glycine buffer (500 µL). The absorbance was then examined using a microplate reader at 570 nm.

### 4.6. Immunofluorescence Staining

Cells were treated with 50 µM DAPT for 72 h. DMSO was used as the vehicle control. The cells were fixed with 10% buffered formalin and washed twice with PBS. The samples were incubated with 10% horse serum for 1 h, then stained with monoclonal mouse anti-human Ki-67 antigen at dilution 1:100 (clone MIB-1, Dako, Glostrup, Denmark) overnight. Biotinylated rabbit anti-mouse antibody was used as the secondary antibody (at dilution 1:500). The samples were incubated with the secondary antibody for 30 min and further stained with Strep-FITC for 30 min. The nuclei were counterstained with DAPI for 10 min. The stained cells were visualized using a fluorescence microscope. Images were randomly captured, and the percentage of Ki67 positive cells per total number of cells in the area was calculated.

### 4.7. Colony Formation Assay

Cells were seeded at 300, 600, or 1,200 cells/well in 24-well plates in a growth medium containing 50 µM DAPT for 14 d, according to previously published protocol [75]. The culture medium was changed every 48 h. The colonies were fixed with cold 100% methanol and stained with 0.1% crystal violet solution for 10 min. Colony sizes were observed using a microscope. The crystal violet was eluted in a 33% acetic acid solution, and its absorbance was measured at 570 nm.

In another experiment, tissue culture plates were coated with agarose in a growth medium (0.6% *w*/*v*). Cells (10 × 10^3^ cells/well) were cultured in 0.3% agarose in growth medium containing 50 µM DAPT or 0.5% DMSO. The next day, the cells were cultured with a 250 µL growth medium with DAPT treatment or DMSO for control. The cells were incubated in a 37 °C, 5% CO_2_ incubator, and the culture media were changed every three days. After culturing for 14 d [75], the visible colonies were captured using bright-field microscopy with a 4X objective lens. The colonies with a diameter ≥ 35 µm were counted manually from 5 random visual fields. The average size of the colonies was quantified using the Zeiss program.

### 4.8. Cell Cycle Analysis

Cells were plated at 2 × 10^5^ cells/well in 6-well plates for 24 h. The cells were incubated with a serum-free medium for serum starvation. After 24 h, the cells were exposed to 50 µM DAPT or 0.5% DMSO for 24 h and 48 h. The cells were collected, fixed in cold 70% ethanol for 15 min, and washed twice with cold PBS. The cells were resuspended in 400 µL FACS buffer. The cell pellets were treated with RNase A to achieve a final concentration of 40 µg/mL and incubated for 30 min at room temperature. Next, propidium iodide solution (20 µL of 1 mg/mL, Sigma-Aldrich, St. Louis, MO, USA) was added and further incubated for 30 min at room temperature in the dark. The stained cells were evaluated using a FACSCalibur (BD Bioscience, San Jose, CA, USA).

### 4.9. Annexin V FITC-PI Apoptosis Assay

Cells (2 × 10^5^ cells/well) were seeded in 6-well plates. The next day, the culture medium was replaced with a growth medium containing 50 µM DAPT or 0.5% DMSO for 48 h. The cells were harvested by trypsinization using 0.25% trypsin/EDTA at 37 °C for 3 min. The growth medium was added to stop trypsin action. Detached cells were then collected by centrifugation for 5 min at 2000 rpm and washed twice with PBS. After centrifugation, a cell pellet was resuspended in Annexin-binding buffer (500 μL), stained with Annexin V-FITC and PI, and then incubated for 10 min in the dark. Subsequently, the stained cells were subjected to examination by flow cytometry analysis.

### 4.10. Transwell Cell Migration

Cells (5 × 10^4^ cells) were resuspended in serum-free medium (200 µL) containing DAPT or DMSO and added to the upper chamber of the transwell (8 μm pore size; Costar). The lower chamber was filled with DMEM (500 µL) supplemented with 20% FBS. After 30 h, the transwell membrane was washed twice with PBS. The non-migrated cells from the upper side of the membrane were gently removed using a cotton swab. The migrated cells on the filter membrane were fixed with cold methanol for 15 min and further stained using 0.1% crystal violet solution for 10 min. The stained cells from 5 random fields were photographed and counted (200× magnification). The number of migrated cells was also measured by eluting the stain in 33% acetic acid solution, and its absorbance was measured at 570 nm [76].

### 4.11. Transwell Invasion Assay

The upper chamber (8 μm pore size; Costar, Sigma-Aldrich, St. Louis, MO, USA) was coated with 30 µL Matrigel (0.32 mg/mL) and allowed to be set for 1 h. Next, a 200 µL cell suspension (5 × 10^4^ cells) in a serum-free medium containing DAPT or DMSO was added to the upper chamber (8 μm pore size; Costar). Then, a culture medium (500 μL) supplemented with 20% FBS was added to the lower chamber. After 24 h, the transwell membrane was washed twice with PBS. The non-invaded cells from the upper side of the membrane were gently removed using a cotton swab. The invaded cells on the filter membrane were fixed with cold methanol for 15 min. The invaded cells were stained using 0.1% crystal violet solution for 10 min. The stained cells from 5 random fields were photographed and counted (200× magnification). The invaded cell numbers were also measured by eluting the stain in 33% acetic acid solution, and its absorbance was measured at 570 nm [76].

### 4.12. Statistical Analysis

Data are presented as mean ± SD. Each dot in the graph represents the individual data value. Statistical analyses were performed using Prism GraphPad version 8.3.1 (GraphPad Software, San Diego, CA, USA). Mann Whitney U test was employed to determine the significant differences between groups. Statistical significance was considered when *p* < 0.05.

## 5. Conclusions

The present study described the dysregulation of Notch signaling-related genes in OSCC biopsies. We showed that inhibiting Notch signaling in vitro significantly reduced cell proliferation, promoted cell cycle arrest, and induced cell apoptosis. Furthermore, cell migration and invasion were also reduced. The role of Notch signaling in OSCC progression and its downstream target genes were explored in our experiments. These results imply the potential application of a Notch signaling inhibitor as a candidate adjuvant chemotherapy in OSCC patients.

## Data Availability

The raw sequencing data were submitted to NCBI’s Gene Expression Omnibus (GEO accession number GSE148919).

## Supplementary Materials

The following supporting information can be downloaded at: https://www.mdpi.com/article/10.3390/ijms23168869/s1.
