# Supplementary material for: A γ-Secretase Inhibitor Attenuates Cell Cycle Progression and Invasion in Human Oral Squamous Cell Carcinoma: An In Vitro Study
Source: Int J Mol Sci. 2022 Aug 9;23(16):8869. doi: 10.3390/ijms23168869 (PMC9408752; doi:10.3390/ijms23168869)
Supplement: Supplementary file 1 [file ijms-23-08869-s001.zip › ijms-1861625-supplementary.pdf]

Supplementary Materials

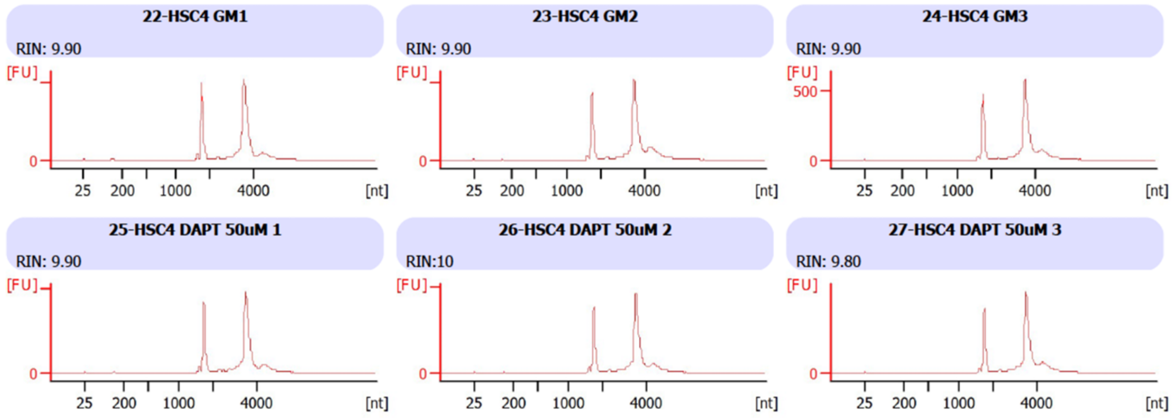

Figure S1: Bioanalyzer traces of submitted total RNA samples.

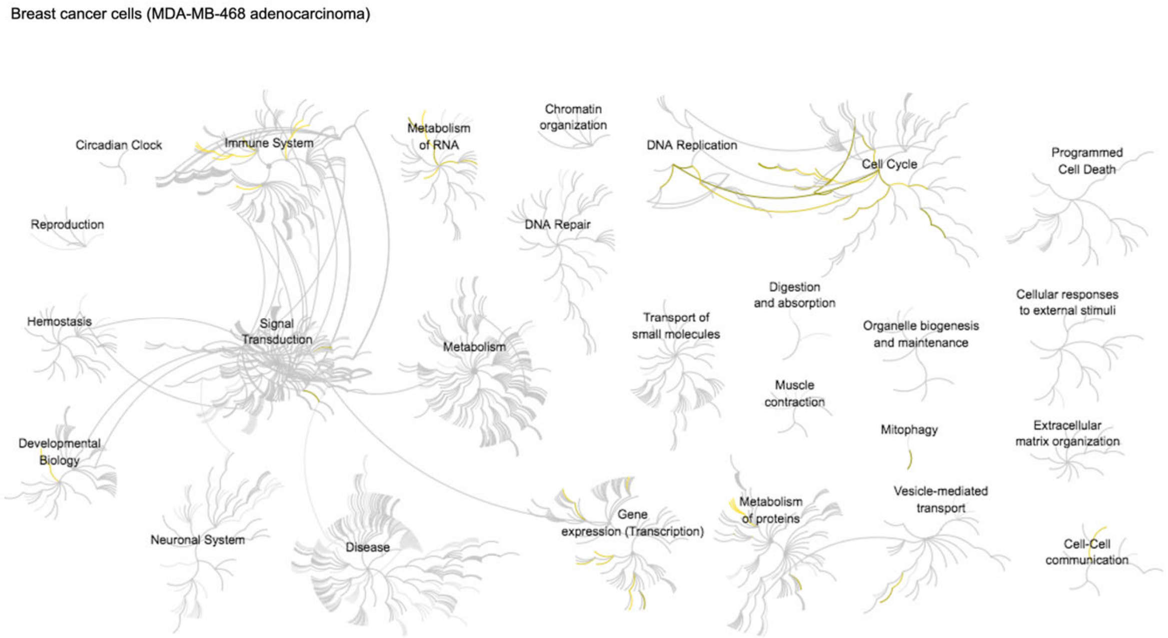

Figure S2: Publicly available dataset of a breast cancer cell line treated with a Notch inhibitor (GSE82298) was downloaded and analysed. Yellow highlights in the diagram shows the dysregulated Reactome pathways in the breast cancer treated with Notch inhibitor.

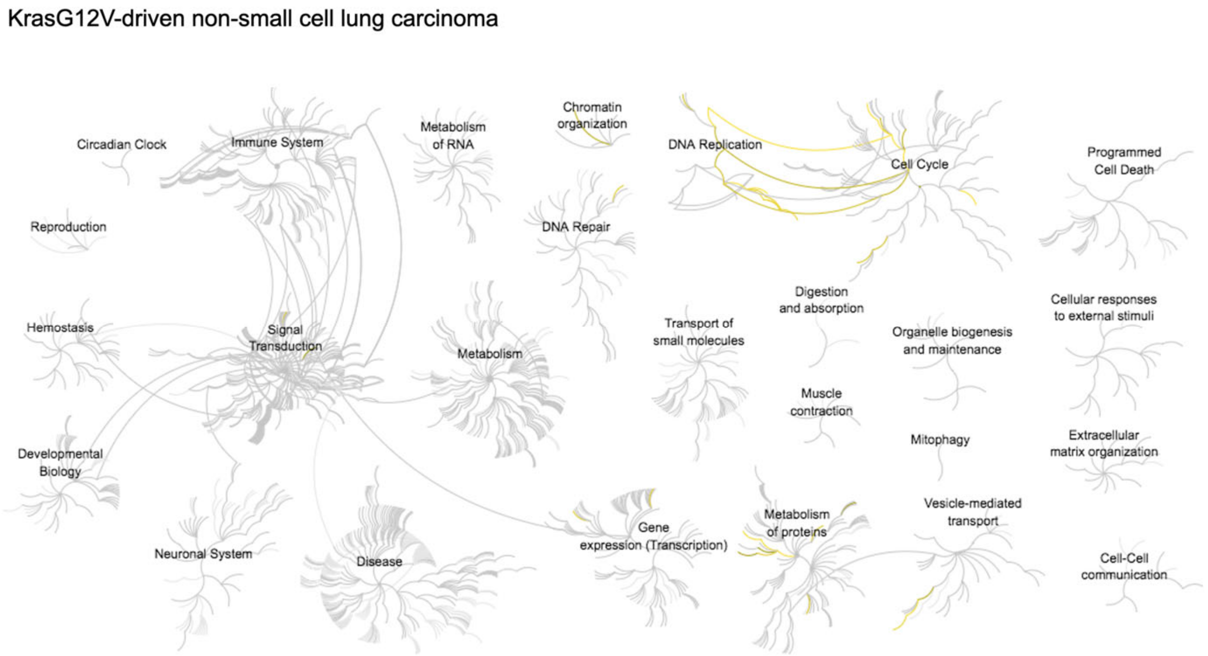

Figure S3: Publicly available dataset of lung cancer cells treated with a Notch inhibitor (GSE38054) were downloaded and analysed. Yellow highlights in the diagram showed the dysregulated Reactome pathways in lung cancer treated with Notch inhibitor.

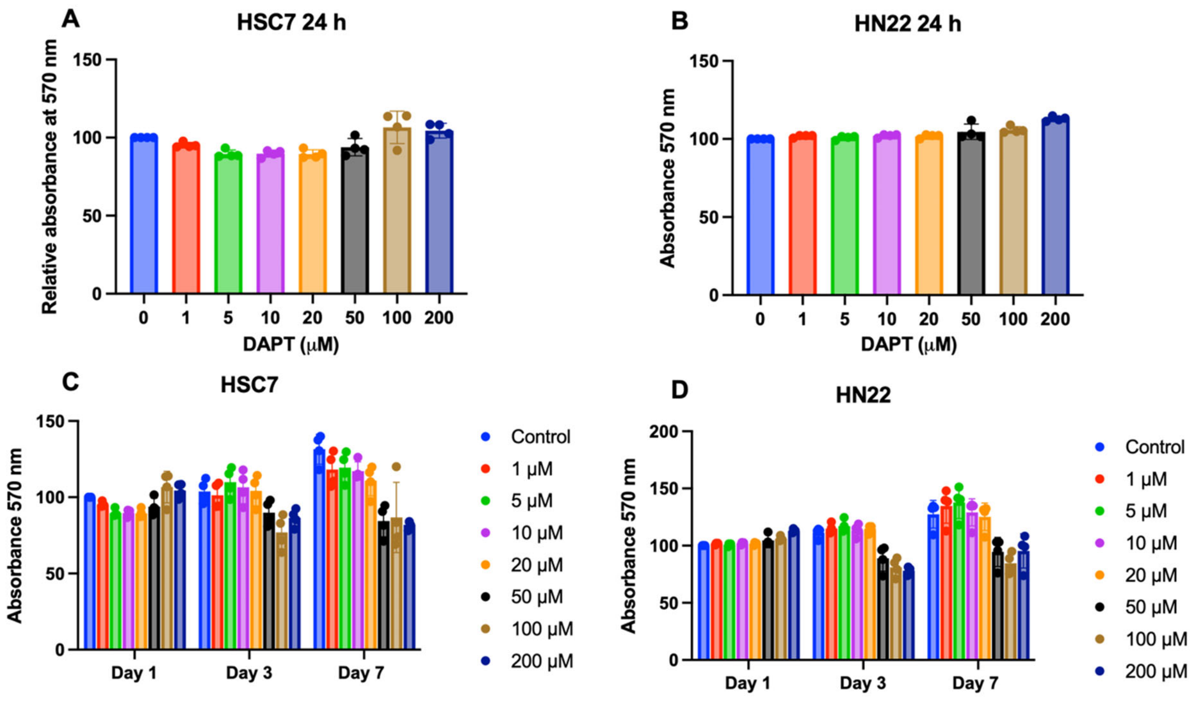

Figure S4: DAPT attenuated HSC-7 and HN22 cell proliferation. Cells were treated with DAPT. DMSO was used as the vehicle control. (A and B) Cell cytotoxicity was evaluated at 24 h using MTT assay. (C and D) Cell proliferation was determined using an MTT assay.

Table S1: Primer sequences.

Gene	Sequences	Aceesion number
CCND1	F: 5'-GGCGGAG GAGAACAAACAGA-3'	NM_053056.3
	R: 5'-A TGGAGGGCGGATTGGAAA-3'	
CCNE2	F: 5'-GCCGAGCGGTAGCTGGTC-3'	NM_057749.2
	R: 5'-GGGCTGCTGCTTAGCTTGTAAA-3'	
E2F1	F: 5' -GAC CCT GAC CTG CTG CTC T- 3'	NM_005225.3
	R: 5' - GGC CAG GTA CTG ATG GTC A-3'	
E2F2	F: 5' -CAA GTT GTG CGA TGC CTG- 3'	NM_004091.4
	R: 5' - TTG GGA ACT CAG GGA CGA -3'	
MCM2	F: 5' - AAT TTC GTC CTG GGT CCT TT -3'	NM_004526.4
	R: 5' - CAC TTT GCC TGG ACT CTC CT-3'	
MCM4	F: 5' - TTC TTT GAC CGT TAC CCT GA-3'	NM_005914.4
	R: 5' - ACA CTT GGC ACT GGA AGA AG-3'	
MCM5	F: 5' - TAT TGC CTA CTG CCG AGT GA-3'	NM_006739.4
	R: 5' - ACT GTC CCT CTC GTG CTG AC-3'	
MCM8	F: 5' - CCA GGC CTA GGA AAA AGT CA-3'	NM_032485.6
	R: 5' - GAG GTG GTC GTG GTG TTA CC-3'	
MCM10	F: 5' - CCG TCT GCA AAA ATC CCC TGA GA-3'	NM_018518.5
	R: 5' - ATG AGC TTT TGG GAT CTG GAG GT-3'	
MMP9	F: 5′- TTT GAC AGC GAC AAG AAG TG-3′	NM_004994.3
	R: 5′- CAG GGC GAG GAC CAT AGA GG-3′	
TIMP1	F: 5′- AGTCAACCAGACCACCTTATACCA-3′	NM_003254.3
	R: 5′- TTTCAGAGCCTTGGAGGAGCTGGTC-3′	
18S	F: 5' - GGCGTCCCCCAACTTCTTA -3'	NR_003286.2
	R: 5' - GGGCATCACAGACCTGTTATT-3'	

Table S2: KEGG pathway enrichment analysis of differentially expressed genes in a g-secretase inhibitor-treated human oral squamous cell carcinoma cell line.

Pathway	Total genes	Hits genes	FDR
Cell cycle	124	53	2.56E-09
DNA replication	36	22	4.71E-07
Ribosome	153	55	8.18E-07
p53 signaling pathway	72	30	4.43E-05
Protein processing in endoplasmic reticulum	165	51	0.000337
HTLV-I infection	219	63	0.000337
Cellular senescence	160	48	0.00111
Prostate cancer	97	32	0.00298
Mismatch repair	23	12	0.00393
Fluid shear stress and atherosclerosis	139	41	0.0045
Lysosome	123	37	0.0055
Antifolate resistance	31	14	0.00558
Parkinson's disease	142	41	0.00558
Pathways in cancer	530	120	0.00558
Carbon metabolism	116	35	0.00558
AGE-RAGE signaling pathway in diabetic complications	100	31	0.00659
Nucleotide excision repair	47	18	0.00659
Biosynthesis of amino acids	75	25	0.00659
Epstein-Barr virus infection	201	53	0.00666
Small cell lung cancer	93	29	0.00752
Viral carcinogenesis	201	52	0.011
HIF-1 signaling pathway	100	30	0.0113
Pathogenic Escherichia coli infection	55	19	0.0154
Chronic myeloid leukemia	76	24	0.0154
Base excision repair	33	13	0.0222
Pyrimidine metabolism	57	19	0.0223
Pancreatic cancer	75	23	0.0246
Proteoglycans in cancer	201	50	0.0246
Fanconi anemia pathway	54	18	0.0246
SNARE interactions in vesicular transport	34	13	0.0246
Hepatitis B	163	42	0.0246
Thyroid hormone signaling pathway	116	32	0.0246
Renal cell carcinoma	69	21	0.0365
Necroptosis	162	41	0.0365

Table S3: Reactome pathway enrichment analysis of differentially expressed genes in a g-secretase inhibitor-treated human oral squamous cell carcinoma cell line.

Pathway	Total genes	Hits genes	FDR
Cell Cycle, Mitotic	411	135	5.25E-12
S Phase	122	57	3.35E-11
DNA strand elongation	31	24	1.51E-10
Cell Cycle	508	152	1.51E-10
Synthesis of DNA	95	47	1.69E-10
DNA Replication	102	49	1.87E-10
G1/S Transition	113	50	4.43E-09
Mitotic G1-G1/S phases	140	57	9.18E-09
Mitotic M-M/G1 phases	266	89	1.37E-08
Extension of Telomeres	24	18	1.33E-07
Telomere C-strand (Lagging Strand) Synthesis	22	17	1.64E-07
Lagging Strand Synthesis	20	15	2.98E-06
Cell Cycle Checkpoints	131	48	1.04E-05
M Phase	233	71	5.16E-05
Gap-filling DNA repair synthesis and ligation in GG-NER	16	12	6.11E-05
Gap-filling DNA repair synthesis and ligation in TC-NER	16	12	6.11E-05
G2/M Checkpoints	48	23	8.93E-05
DNA Replication Pre-Initiation	80	32	0.000109
M/G1 Transition	80	32	0.000109
Processive synthesis on the lagging strand	15	11	0.000193
Repair synthesis of patch ~27-30 bases long by DNA polymerase	15	11	0.000193
Repair synthesis for gap-filling by DNA polymerase in TC-NER	15	11	0.000193
Activation of ATR in response to replication stress	41	20	0.000235
Activation of the pre-replicative complex	32	17	0.000275
Unwinding of DNA	11	9	0.000318
Unfolded Protein Response	66	27	0.000318
G1/S-Specific Transcription	16	11	0.000424
Metabolism of proteins	689	162	0.000565
Removal of the Flap Intermediate	14	10	0.000565
Leading Strand Synthesis	14	10	0.000565
Polymerase switching	14	10	0.000565
Polymerase switching on the C-strand of the telomere	14	10	0.000565
Cyclin E associated events during G1/S transition	65	26	0.000602
E2F mediated regulation of DNA replication	35	17	0.000917

Pathway	Total genes	Hits genes	FDR
Removal of licensing factors from origins	71	27	0.00112
Regulation of DNA replication	71	27	0.00112
Mitotic Prometaphase	127	41	0.00116
Ribosomal scanning and start codon recognition	91	32	0.00128
G0 and Early G1	27	14	0.00159
Antiviral mechanism by IFN-stimulated genes	69	26	0.00159
ISG15 antiviral mechanism	69	26	0.00159
Switching of origins to a post-replicative state	69	26	0.00159
Orc1 removal from chromatin	69	26	0.00159
Separation of Sister Chromatids	186	54	0.00173
Cyclin A:Cdk2-associated events at S phase entry	66	25	0.00191
Processive synthesis on the C-strand of the telomere	11	8	0.00255
Antigen Presentation: Folding, assembly and peptide loading of class I MHC	25	13	0.00255
Mitotic Anaphase	198	56	0.00256
Mitotic Metaphase and Anaphase	199	56	0.00292
Telomere Maintenance	72	26	0.00314
Translation initiation complex formation	92	31	0.00317
DNA Repair	117	37	0.00324
Folding of actin by CCT/TriC	9	7	0.00334
GTP hydrolysis and joining of the 60S ribosomal subunit	201	56	0.00355
Activation of the mRNA upon binding of the cap-binding complex and eIFs, and subsequent binding to 43S	93	31	0.00366
Resolution of Sister Chromatid Cohesion	118	37	0.00366
Formation of tubulin folding intermediates by CCT/TriC	23	12	0.0038
SCF(Skp2)-mediated degradation of p27/p21	58	22	0.00395
Metabolism of RNA	339	85	0.00436
Formation of the ternary complex, and subsequently, the 43S complex	83	28	0.00556
3' -UTR-mediated translational regulation	201	55	0.00588
L13a-mediated translational silencing of Ceruloplasmin expression	201	55	0.00588
Activation of Chaperones by IRE1alpha	49	19	0.00721
Removal of the Flap Intermediate from the C-strand	10	7	0.00783

Pathway	Total genes	Hits genes	FDR
Metabolism of nucleotides	81	27	0.00821
Eukaryotic Translation Initiation	209	56	0.00859
Cap-dependent Translation Initiation	209	56	0.00859
Interferon Signaling	173	48	0.00921
Assembly of collagen fibrils and other multimeric structures	54	20	0.00941
G1/S DNA Damage Checkpoints	62	22	0.00984
Global Genomic NER (GG-NER)	36	15	0.011
Chaperonin-mediated protein folding	51	19	0.0114
Formation of a pool of free 40S subunits	189	51	0.0118
Assembly of the pre-replicative complex	63	22	0.012
DNA replication initiation	6	5	0.0151
Telomere C-strand synthesis initiation	6	5	0.0151
Cooperation of Prefoldin and TriC/CCT in actin and tubulin folding	30	13	0.0151
APC/C-mediated degradation of cell cycle proteins	89	28	0.0152
Regulation of mitotic cell cycle	89	28	0.0152
Phosphorylation of proteins involved in G1/S transition by active Cyclin E:Cdk2 complexes	4	4	0.0162
Cytosolic tRNA aminoacylation	24	11	0.0206
Cytokine Signaling in Immune system	286	70	0.0232
Gluconeogenesis	35	14	0.0232
Metabolism of mRNA	317	76	0.0267
p53-Dependent G1/S DNA damage checkpoint	59	20	0.0273
p53-Dependent G1 DNA Damage Response	59	20	0.0273
Type I hemidesmosome assembly	12	7	0.0274
Chromosome Maintenance	124	35	0.0294
Association of TriC/CCT with target proteins during biosynthesis	29	12	0.033
Prefoldin mediated transfer of substrate to CCT/TriC	29	12	0.033
Protein folding	56	19	0.033
Activation of APC/C and APC/C:Cdc20 mediated degradation of mitotic proteins	77	24	0.0342
Activation of NIMA Kinases NEK9, NEK6, NEK7	7	5	0.0373
Nucleotide Excision Repair	53	18	0.0408
Glucose metabolism	70	22	0.0443
Metabolism of folate and pterines	10	6	0.0453

Table S4: Gene ontology (Biological process) enrichment analysis of differentially expressed genes in a g-secretase inhibitor-treated human oral squamous cell carcinoma cell line.

Pathway	Total genes	Hits genes	FDR
S phase	153	72	3.35E-15
S phase of mitotic cell cycle	144	67	5.07E-14
Viral reproductive process	597	176	1.82E-12
Interphase	443	136	6.86E-11
Interphase of mitotic cell cycle	435	133	1.41E-10
Mitotic cell cycle	968	246	4.05E-10
Cellular component disassembly	310	102	4.05E-10
Protein complex disassembly	167	62	2.63E-08
Viral reproduction	803	204	2.63E-08
Cellular protein complex disassembly	160	60	2.80E-08
G1/S transition of mitotic cell cycle	209	72	3.72E-08
Macromolecular complex disassembly	189	67	3.72E-08
Cell cycle arrest	428	122	6.92E-08
Cell cycle phase	1070	255	1.12E-07
Viral infectious cycle	241	78	1.56E-07
Regulation of cell cycle	886	216	1.98E-07
Regulation of mitotic cell cycle	351	103	1.98E-07
Cell cycle checkpoint	281	86	4.14E-07
Translational initiation	205	68	4.14E-07
ER_nucleus signaling pathway	111	44	4.41E-07
DNA_dependent DNA replication	121	46	9.15E-07
Negative regulation of cell cycle	520	135	3.11E-06
Intracellular protein transport	793	191	3.11E-06
Protein targeting to membrane	158	54	3.52E-06
Endoplasmic reticulum unfolded protein response	93	37	4.58E-06
Cell division	507	130	1.01E-05
Mitotic cell cycle checkpoint	149	50	1.73E-05
Mitosis	420	110	2.54E-05
DNA replication initiation	30	17	2.71E-05
Interaction with host	426	111	2.77E-05
DNA replication	346	93	4.89E-05
Cell cycle process	1420	304	5.00E-05
M phase of mitotic cell cycle	447	114	5.58E-05
Positive regulation of cell cycle	113	39	0.000108
RNA catabolic process	256	72	0.000112
Cellular macromolecule catabolic process	849	193	0.000113
Cell cycle	1860	383	0.000146
Sister chromatid segregation	57	24	0.000146
Cellular response to stress	1620	339	0.000165
Response to ionizing radiation	112	37	0.00049
Spindle organization	92	32	0.000525
Mitotic sister chromatid segregation	54	22	0.000589
DNA integrity checkpoint	152	46	0.000634

Pathway	Total genes	Hits genes	FDR
Chromosome segregation	174	51	0.000634
Intrinsic apoptotic signaling pathway	135	42	0.000651
Macromolecule catabolic process	1070	230	0.000697
Macromolecule catabolic process	1070	230	0.000697
Negative regulation of apoptotic process	679	154	0.000855
Negative regulation of apoptotic process	679	154	0.000855
Androgen receptor signaling pathway	67	25	0.000855
Negative regulation of programmed cell death	691	155	0.00141
DNA damage checkpoint	143	42	0.00242
Protein targeting	545	125	0.00245
DNA repair	538	123	0.00311
G1 phase	49	19	0.00337
Signal transduction in response to DNA damage	129	38	0.00424
Response to hypoxia	245	63	0.00448
Organelle localization	189	51	0.00472
Protein folding	241	62	0.00475
DNA damage response, signal transduction by p53 class mediator	117	35	0.0049
Intracellular steroid hormone receptor signaling pathway	113	34	0.00516
G1 phase of mitotic cell cycle	47	18	0.00519
M phase	671	147	0.00526
Negative regulation of transcription from RNA polymerase II promoter	552	124	0.00526
G2/M transition of mitotic cell cycle	150	42	0.00596
Aging	197	52	0.00646
Response to abiotic stimulus	876	185	0.00685
Response to DNA damage stimulus	862	182	0.00756
DNA recombination	247	62	0.00812
Chromosome condensation	34	14	0.0085
Intracellular transport	1510	301	0.0085
Cellular component disassembly involved in execution phase of apoptosis	78	25	0.00901
Chromosome organization	878	184	0.00958
Golgi vesicle transport	206	53	0.0098
Regulation of hydrolase activity	843	177	0.0105
Positive regulation of binding	79	25	0.0105
Apoptotic signaling pathway	261	64	0.0115
Regulation of binding	189	49	0.0119
Establishment of organelle localization	125	35	0.0139
Response to oxidative stress	279	67	0.0153
Protein export from nucleus	48	17	0.0158
Regulation of mitosis	99	29	0.0158
Neuron apoptotic process	169	44	0.0178

Pathway	Total genes	Hits genes	FDR
Positive regulation of cysteine_type endopeptidase activity involved in apoptotic process	118	33	0.0181
Body fluid secretion	74	23	0.0192
Base_excision repair	45	16	0.0195
Negative regulation of protein metabolic process	540	117	0.0216
Regulation of neuron apoptotic process	150	39	0.0299
Positive regulation of I_kappaB kinase/NF_kappaB cascade	150	39	0.0299
Intrinsic apoptotic signaling pathway in response to DNA damage	51	17	0.03
Macromolecular complex assembly	1120	223	0.0388
Intra_Golgi vesicle_mediated transport	32	12	0.0391
Microtubule cytoskeleton organization	337	76	0.0395
Protein homooligomerization	226	54	0.0395
Apoptotic mitochondrial changes	83	24	0.0398
Regulation of organelle organization	589	124	0.0413
Nuclear export	139	36	0.0413
Gland development	303	69	0.043
Response to toxin	130	34	0.043
Protein catabolic process	644	134	0.0437
Regulation of translation	228	54	0.0445
Response to radiation	345	77	0.0451
Regulation of cyclin_dependent protein kinase activity	89	25	0.0465
Positive regulation of hydrolase activity	497	106	0.0465
Protein transport	1400	272	0.0473
Regulation of chromosome organization	122	32	0.0482
Nucleocytoplasmic transport	388	85	0.0482
Regulation of I_kappaB kinase/NF_kappaB cascade	210	50	0.05

Table S5: Gene ontology (Cellular Component) enrichment analysis of differentially expressed genes in a g-secretase inhibitor-treated human oral squamous cell carcinoma cell line.

Pathway	Total genes	Hits genes	FDR
Cytosol	2660	592	1.93E-19
Ribosomal subunit	154	56	3.89E-08
Ribonucleoprotein complex	681	164	5.12E-07
Nucleoplasm	1820	371	1.53E-06
Nuclear lumen	2690	521	1.59E-06
Cytosolic part	204	63	1.59E-06
Membrane_enclosed lumen	3440	648	1.59E-06
Organelle lumen	3380	637	1.87E-06
Chromosome	784	178	3.50E-06
Condensed chromosome	193	59	4.08E-06
Spindle	261	72	1.56E-05
Nuclear chromosome	320	84	1.74E-05
Nuclear chromosome part	273	72	7.17E-05
Ribosome	249	67	7.17E-05
Chromosomal part	670	149	7.17E-05
Microtubule organizing center	543	125	7.17E-05
Nucleolus	652	145	8.92E-05
Centrosome	412	99	9.36E-05
Small ribosomal subunit	72	26	0.000243
Chromatin	326	80	0.000278
PML body	77	27	0.000278
Nuclear part	3330	604	0.000278
Replication fork	50	20	0.000327
Nuclear membrane	207	55	0.000435
Macromolecular complex	4800	842	0.000442
Perinuclear region of cytoplasm	475	105	0.00134
Non_membrane_bounded organelle	3940	696	0.00134
Intracellular non_membrane_bounded organelle	3940	696	0.00134
Nuclear envelope	387	88	0.00148
Nuclear body	295	70	0.00172
Nucleoplasm part	910	182	0.0022
Nuclear chromatin	159	42	0.00279
Cell cortex	195	48	0.00612
Chromosome, centromeric region	198	48	0.00842
Microtubule cytoskeleton	1120	213	0.00927
Microtubule	352	77	0.00939
Spindle pole	101	28	0.00965
Organelle outer membrane	156	39	0.0113
Pore complex	103	28	0.0124
U12_type spliceosomal complex	24	10	0.0124
Nuclear replication fork	28	11	0.0129
Outer membrane	163	40	0.013
Microtubule organizing center part	100	27	0.015
Transcription factor complex	303	66	0.0178

Pathway	Total genes	Hits genes	FDR
Integral to endoplasmic reticulum membrane	118	30	0.0226
Mitochondrial outer membrane	133	33	0.0226
Spindle microtubule	43	14	0.0234
Protein complex	4050	693	0.0238
Golgi membrane	605	119	0.0255
Tight junction	105	27	0.0267
Endoplasmic reticulum membrane	872	165	0.0275
Nuclear pore	86	23	0.0277
Nuclear matrix	87	23	0.0316
Condensed nuclear chromosome	73	20	0.0333
Apical junction complex	123	30	0.0354
Kinetochore	149	35	0.0361
Spliceosomal complex	161	37	0.0417
Eukaryotic translation initiation factor 3 complex	17	7	0.0426
Nuclear outer membrane_endoplasmic reticulum membrane network	894	166	0.0469

Table S6: Gene ontology (Molecular Function) enrichment analysis of differentially expressed genes in a g-secretase inhibitor-treated human oral squamous cell carcinoma cell line.

Pathway	Total genes	Hits genes	FDR
Structural constituent of ribosome	167	58	1.47E-06
RNA binding	976	205	0.00434
Damaged DNA binding	46	19	0.00434
Chromatin binding	338	83	0.00434
Enzyme binding	1200	244	0.00434
Protein C_terminus binding	160	44	0.0136
DNA_dependent ATPase activity	80	26	0.014
Structural constituent of cytoskeleton	78	25	0.0202
Nucleotide binding	2470	458	0.0216
Kinase binding	418	93	0.0288
ATP_dependent DNA helicase activity	36	14	0.0297
ATP binding	1490	285	0.0297
Single_stranded DNA binding	65	21	0.0297
Adenyl ribonucleotide binding	1530	291	0.0297
Protein kinase binding	376	84	0.0297
Ras GTPase binding	125	34	0.0305
Adenyl nucleotide binding	1530	291	0.0305
Structural molecule activity	666	137	0.0342
Structure_specific DNA binding	242	57	0.0401
Magnesium ion binding	188	46	0.0446
Steroid hormone receptor binding	69	21	0.0446
GTPase binding	150	38	0.0497
